# Supplementary material for: Mapping vaccination coverage to explore the effects of delivery mechanisms and inform vaccination strategies
Source: Nat Commun. 2019 Apr 9;10:1633. doi: 10.1038/s41467-019-09611-1 (PMC6456602; doi:10.1038/s41467-019-09611-1)
Supplement: Supplementary file 1 — Supplementary Information [file 41467_2019_9611_MOESM1_ESM.pdf]

Mapping vaccination coverage to explore the effects of delivery mechanisms and inform vaccination strategies

Utazi CE, Thorley J, Alegana V, Ferrari M, Takahashi S, Metcalf C, Lessler J, Cutts FT and Tatem AJ

Supplementary Information

This document accompanies the main paper. It contains the supplementary methods and supplementary tables and figures referenced in the paper.

Supplementary Methods

Here, we provide further details of the models used in mapping MCV and DTP coverage. The coregionalization approach for the cross-covariance function $\boldsymbol{C}_w$ in the multivariate model for DTP is based on the linear transformation $\boldsymbol{w}(\boldsymbol{s}) = \boldsymbol{A}(\boldsymbol{s})\widetilde{\boldsymbol{w}}(\boldsymbol{s})$, where $\boldsymbol{A}(\boldsymbol{s})$ is a non-singular (possibly) space-varying matrix transform, $\widetilde{\boldsymbol{w}}(\boldsymbol{s}) = \left(\widetilde{w}_1(\boldsymbol{s}), \widetilde{w}_2(\boldsymbol{s}), \widetilde{w}_3(\boldsymbol{s})\right)$ and $\widetilde{w}_j(\boldsymbol{s})$ is an independent spatial process with unit variance, i.e. var $\left(\widetilde{w}_j(\boldsymbol{s})\right) = 1$, and correlation function $\rho_j(\boldsymbol{s}, \boldsymbol{s}')$. Thus, the cross-covariance matrix for $\widetilde{\boldsymbol{w}}(\boldsymbol{s})$, $\boldsymbol{C}_{\widetilde{w}}(\boldsymbol{s}, \boldsymbol{s}')$, is diagonal with jth diagonal element given by $\rho_j(\boldsymbol{s}, \boldsymbol{s}')$, yielding a valid nonstationary cross-covariance $\boldsymbol{C}_w(\boldsymbol{s}, \boldsymbol{s}') = \boldsymbol{A}(\boldsymbol{s})\boldsymbol{C}_{\widetilde{w}}(\boldsymbol{s}, \boldsymbol{s}')\boldsymbol{A}(\boldsymbol{s}')'$ for $\boldsymbol{w}(\boldsymbol{s})$ (see [1]). As in the model for measles, we modelled $\rho_j(\boldsymbol{s}, \boldsymbol{s}')$ using the exponential correlation function given by $\rho_j(\boldsymbol{s}, \boldsymbol{s}') = \exp(-\phi_j \ \|\boldsymbol{s} - \boldsymbol{s}'\|)$, where ϕ_j is a spatial decay parameter and $\|.\|$ denotes the Euclidean distance. Under the assumption of stationarity, the linear transformation is independent of space, so that $\boldsymbol{A}(\boldsymbol{s}) = \boldsymbol{A}$ and $\boldsymbol{C}_w(\boldsymbol{s}, \boldsymbol{s}') = \boldsymbol{A}\boldsymbol{C}_{\widetilde{w}}(\boldsymbol{s}, \boldsymbol{s}')\boldsymbol{A}'$; which also implies that $\boldsymbol{C}_w(\boldsymbol{s}, \boldsymbol{s}) = \boldsymbol{C}_w(\boldsymbol{0}) = \boldsymbol{A}\boldsymbol{A}'$ (since $\boldsymbol{C}_{\widetilde{w}}(\boldsymbol{s}, \boldsymbol{s}) = I_3$). We assign an inverse-Wishart prior $(3, \boldsymbol{I}_3)$ to the cross-covariance matrix, $\boldsymbol{A}\boldsymbol{A}'$; see [2]. To complete the Bayesian specification, we place the weakly informative prior: Normal $(\boldsymbol{0}, 10^3\boldsymbol{I})$ on $\boldsymbol{\beta}_j$. For ϕ_j, we use country-specific Uniform (a_ϕ, b_ϕ) prior distributions. For DRC and Ethiopia, we set $a_\phi = 0.166$ and b_ϕ =1.11, corresponding to an effective range of between 300 and 2000 km. For Mozambique, Nigeria and Cambodia, we set $a_\phi = 0.475, 0.33, 0.66$ and $b_\phi = 3.3, 1.11, 6.6$, respectively. These yield effective ranges of between 100 and 700 km for Mozambique, 300 and 1000 km for Nigeria, and 50 and 504 km for Cambodia. (The effective range is the distance at which spatial correlation drops to 0.05, calculated here as $-\log(0.05)/\phi_j$ in decimal degrees.) These uniform priors were chosen due to the need to include moderate levels of correlation in the models and in some cases, relative to the geographical extents of the countries. We note that for comparability, these uniform priors were also placed on the spatial decay parameters in the respective univariate models for MCV.

Additional information about the multivariate model can be found in [1] and [2]. To ease the computational burden associated with fitting these models, particularly the multivariate model, we use the predictive process model specification in spBayes for both MCV and DTP1-3. The predictive process model projects the spatial process - $\boldsymbol{w} = (w(\boldsymbol{s}_1), \dots, w(\boldsymbol{s}_n))$ for MCV and $\boldsymbol{W} = \left(\boldsymbol{w}^T(\boldsymbol{s}_1), \dots, \boldsymbol{w}^T(\boldsymbol{s}_n)\right)$ for DTP1-3 – to a lower-dimensional process that is generated by the realizations of the original process at a specified set of locations known as knot locations. Following pilot runs, we chose 150 knot locations (from the DHS cluster or data locations) for Nigeria, 200 for DRC and 100 for Ethiopia, Cambodia and Mozambique. The knot locations were determined using a space-filling design algorithm [3]. As in [4], the MCMC algorithms were run for 90,000 iterations after a burn-in of 10,000 iterations. Convergence was assessed by examining the trace plots of the parameters and through other convergence diagnostics such as the Geweke statistic [5].

To evaluate the performance of the fitted models (both MCV and DTP1-3) for out-of-sample prediction, we adopted the Monte Carlo method of cross-validation repeated 10 times with 10% of the DHS cluster-level data set aside for validation each time. As in [4], the following model evaluation metrics were computed (and averaged) using the observed (p_i) and

predicted ($\hat{p}_i$) probabilities for the validation cluster locations: percentage bias (%bias $=$ $100 \times \sum_{i=1}^{m}(\hat{p}_i - p_i)/\sum_{i=1}^{m} p_i$), validation mean square error (VMSE= $\sum_i(\hat{p}_i - p_i)^2/m$). Also, the achieved coverage of the 95% prediction intervals was calculated as: $(100 \times \sum_i I(l_i \leq y_i \leq u_i)/m)$, where l_i and u_i denote the lower and upper limits of the prediction interval for the number of children vaccinated at the i th validation location, y_i the corresponding observed number of children vaccinated, $I(.)$ is an indicator function and m is the number of validation locations. The closer the values of %bias and VMSE are to zero, the better the predictions. Similarly, the closer the achieved coverage is to its true value (i.e. 95%), the better the predictions. Finally, the R^2 values of the fitted models during cross-validation were calculated as the square of the sample (or Pearson's) correlation coefficient between the observed and predicted probabilities. Note that the %bias formula given here can also be written as %bias = $\frac{100}{m} \times \sum_{i=1}^{m}[(\hat{p}_i - p_i)/\bar{p}]$. The division by $\bar{p}$ instead of p_i was due to the fact that in the latter case, %bias would be undefined when $p_i = 0$ as was the case in some cluster locations.

Supplementary Table 1: Selected covariates for modeling MCV and DTP1-3

Country	MCV	DTP1-3
Nigeria	Poverty, Aridity, Nighttime lights, Travel time (to major cities), EVI	Poverty, Aridity, Travel time, EVI
Ethiopia	Dist. to urban areas, Population density, Travel time, Dist. to water, Dist. to rail	Dist. to urban areas, Travel time, MODIS net primary production, Pig density
DRC	Dist. to urban areas, Cattle density, Temperature, Pig density, Aridity	Dist. to urban areas, Temperature, Cattle density, Slope, MODIS net primary production
Cambodia	Travel time, Population density, Dist. to residential areas, Dist. to infrastructures	Travel time, MODIS net primary production, Slope, Dist. to places
Mozambique	Travel time, Precipitation, Evapotranspiration, MODIS net primary production	Precipitation, Dist. to highways, Slope, Evapotranspiration

Supplementary Table 2: Description of selected covariates for modeling MCV and DTP1-3

**Covariate**	**Description**	**Unit**	**Source**
Poverty	Proportion of people living in poverty, as defined by $2.00 / day.	-	Tatem, A. J., D. Weiss, and C. Pezzulo. "Pilot high resolution poverty maps." *Southampton: Oxford Uo* (2013)
Aridity	Aridity Index (AI) = MAP / MAE where: MAP = Mean Annual Precipitation and MAE = Mean Annual Potential EvapoTranspiration. Annual average for the 1950- 2000 period.	Index values increase for more humid conditions, and decrease with more arid conditions	CGIAR-CSI Global-Aridity and Global-PET Database www.cgiar-csi.org
Global Potential Evapo-Transpiration	Potential Evapo-Transpiration (PET) is a measure of the ability of the atmosphere to remove water through evapotranspiration. Annual average for the 1950- 2000 period.	mm/month	CGIAR-CSI Global-Aridity and Global-PET Database www.cgiar-csi.org
MODIS net primary production	A measure of the growth of the terrestrial vegetation. Annual NPP is derived from the sum of the 45, 8-day Net Photosynthesis (PSN) products from the given year.	g C m^2	Running, S., Mu, Q., Zhao, M. (2015). MOD17A3H MODIS/Terra Net Primary Production Yearly L4 Global 500m SIN Grid V006. NASA EOSDIS Land Processes DAAC.
Enhanced Vegetation Index (EVI)	A measure of vegetation canopy greenness, a combined property of leaf area, chlorophyll and canopy structure.	Index values increase with canopy greenness	Didan, K. (2015). *MOD13A3 MODIS/Terra vegetation Indices Monthly L3 Global 1km SIN Grid V006*. NASA EOSDIS LP DAAC.
Temperature	Average for the 1970-2000 period.	°C	Fick, S.E. and Hijmans, R.J. (2017) WorldClim 2: New spatial resolution climate surfaces for global land areas. International journal of Climatology. www.worldclim.org
Precipitation	Average for the 1970-2000 period.	mm	As above.
Slope	Slope in degrees, derived from elevation data.	°	US Geological Survey (USGS) http://eros.usgs.gov/elevation-products
Cattle density	Livestock densities – global distribution of cattle.	heads per sq. km	Gridded Livestock of the World v2.0, Robinson T.P., Wint, G.R.W., Conchedda, G., Van Boeckel, T.P., Ercoli,

			V., Palamara, E., Cinardi, G., D'Aietti, L., Hay, S.I and Gilbert, M. (2014) Mapping the global distribution of livestock. PLoS ONE 9(5):e96084. doi:10.1371/journal.pone.0096084.
Pig density	Livestock densities – global distribution of pigs.	heads per sq. km	As above.
Travel time	Travel time to major cities of >50,000 people in the year 2000.	Minutes	**Nelson, A. (2008)** Estimated travel time to the nearest city of 50,000 or more people in year 2000. Global Environment Monitoring Unit - Joint Research Centre of the European Commission, Ispra Italy. Available at http://forobs.jrc.ec.europa.eu/products/gam/
Night-time lights	Land-based night-time lights.	Nano-Watts/(sqcm*sr)	NOAA VIIRS ngdc.noaa.gov/eog
Distance to urban areas	Derived from global maps of urban extent based on Moderate Resolution Imaging Spectroradiometer (MODIS) 500m satellite data.	Distance (m)	Schneider, A., Friedl, M., Potere, D., 2009, A new map of global urban extent from MODIS data. Environmental Research Letters, vol. 4, article 044003.
Distance to water, rail, residential areas, infrastructures, places, highways	Derived from OSM input data.	Distance (m)	Input data from Open Street map (2016) www.openstreetmap.org
Population density	Estimates of numbers of people per grid square, with national totals adjusted to match UN population division estimates.	Estimated persons per grid square	WorldPop Project www.worldpop.org

Supplementary Table 3. Estimates of the parameters of the fitted models for Nigeria. Reported are the posterior means, standard deviations (SD) and quantiles (2.5%, 50% and 97.5%) of the regression coefficients and the parameters of the spatial random effects.

Parameter	Mean	SD	2.5%	50%	97.5%
MCV (9-59 months)					
(Intercept)	1.6556	0.2706	1.1776	1.6502	2.1967
Poverty	-2.8564	0.2717	-3.4510	-2.8400	-2.1844
Aridity	4.7×10^{-6}	2.03×10^{-5}	-4.23×10^{-5}	-4.42×10^{-6}	3.28×10^{-5}
log(Nighttime lights)	0.4214	0.0512	0.3219	0.4223	0.5212
log(Travel time)	-0.1419	0.0326	-0.2061	-0.1376	-0.0799
EVI	2.8967	0.6237	1.7217	2.8621	4.2550
Partial sill (σ^2)	2.1090	0.3829	1.5018	2.0549	2.9940
Spatial decay (ϕ)*	0.9174	0.1320	0.6161	0.9406	1.1005
DTP1 (0-59 months)					
(Intercept)	1.2947	0.2790	0.7771	1.2775	1.8385
Poverty	-1.1834	0.3457	-1.7859	-1.1586	-0.5897
Aridity	0.0001	1.15×10^{-5}	3.45×10^{-5}	0.0001	0.0001
log(Travel time)	-0.2082	0.0216	-0.2493	-0.2065	-0.1670
EVI	0.7272	0.4168	0.0543	0.6861	1.6018
Spatial decay (ϕ_1)*	0.8650	0.1757	0.4525	0.8995	1.0997
DTP2 (0-59 months)					
(Intercept)	0.3135	0.3410	-0.2429	0.3807	0.8653
Poverty	-0.3941	0.2978	-0.9670	-0.3919	0.1488
Aridity	0.0001	1.10×10^{-5}	4.45×10^{-5}	0.0001	0.0001
log(Travel time)	-0.2266	0.0215	-0.2654	-0.2269	-0.1837
EVI	0.5815	0.5051	-0.3773	0.5807	1.5039
Spatial decay (ϕ_2)*	0.4370	0.1357	0.3324	0.3884	0.8839
DTP3 (0-59 months)					
(Intercept)	-0.0392	0.3622	-0.6451	0.0250	0.5915
Poverty	-0.4324	0.2831	-0.8896	-0.4693	0.3092
Aridity	0.0001	1.02×10^{-5}	3.45×10^{-5}	0.0001	0.0001
log(Travel time)	-0.2313	0.0217	-0.2724	-0.2328	-0.1853
EVI	0.6473	0.4003	-0.0997	0.6325	1.4254
Spatial decay (ϕ_3)*	0.5648	0.2156	0.3344	0.4860	1.0637
Cross covariance parameters $\boldsymbol{C}_w$ for DTP1-3**					
$\boldsymbol{C}_{w(DTP1)}$	3.7410	0.9755	2.5107	3.4862	6.4356
$\boldsymbol{C}_{w(DTP1,DTP2)}$	3.5767	0.9588	2.3376	3.3349	6.3213
$\boldsymbol{C}_{w(DTP1,DTP3)}$	3.5011	0.8954	2.3668	3.2804	5.9156
$\boldsymbol{C}_{w(DTP2)}$	3.5211	0.9704	2.2585	3.3048	6.3656
$\boldsymbol{C}_{w(DTP2,DTP3)}$	3.4197	0.8977	2.2357	3.2237	5.8537
$\boldsymbol{C}_{w(DTP3)}$	3.4391	0.8554	2.3490	3.2494	5.6989

*These correspond to effective ranges of 363 km, 385 km, 761 km and 588 km, respectively.

**The estimated values here correspond to correlations of 0.99, 0.98 and 0.98 between DTP1 and 2, DTP1 and 3 and DTP2 and 3, respectively.

Supplementary Table 4. Estimates of the parameters of the fitted models for Ethiopia. Reported are the posterior means, standard deviations (SD) and quantiles (2.5%, 50% and 97.5%) of the regression coefficients and the parameters of the spatial random effects.

Parameter	Mean	SD	2.5%	50%	97.5%
MCV (9-59 months)					
(Intercept)	1.7920	0.7300	-0.0642	1.7594	2.9073
log(Dist. to urban areas)	-0.2034	0.0451	-0.2856	-0.2083	-0.1082
log(Population density)	0.1212	0.0558	0.0084	0.1222	0.2326
log(Travel time)	0.0538	0.0701	-0.1133	0.0465	0.1918
log(Dist. to water)	-0.1194	0.0419	-0.1983	-0.1193	-0.0423
log(Dist. to rail	0.0397	0.0717	-0.0817	0.0328	0.1575
Partial sill (σ^2)	1.5968	0.3743	0.9951	1.5598	2.4659
Spatial decay (ϕ)*	0.9487	0.1351	0.6163	0.9845	1.1037
DTP1 (0-59 months)					
(Intercept)	5.9386	0.5635	4.7910	5.9074	7.1558
log(Dist. to urban areas)	-0.3463	0.0434	-0.4346	-0.3424	-0.2716
log(Travel time)	-0.2021	0.0514	-0.3078	-0.1997	-0.1032
log(MODIS net primary production)	0.2962	0.1577	-0.0110	0.2953	0.6021
log(Pig density)	1.2492	0.3601	0.6359	1.2251	2.0065
Spatial decay (ϕ_1)*	0.3027	0.1161	0.1702	0.2675	0.5804
DTP2 (0-59 months)					
(Intercept)	5.4473	0.5300	3.9971	5.5488	6.1894
log(Dist. to urban areas)	-0.3555	0.0430	-0.4554	-0.3560	-0.2811
log(Travel time)	-0.2157	0.0508	-0.3172	-0.2180	-0.1072
log(MODIS net primary production)	0.1242	0.1492	-0.1655	0.1233	0.4169
log(Pig density)	1.1277	0.3170	0.4977	1.1318	1.7928
Spatial decay (ϕ_2)*	0.3711	0.2443	0.1690	0.2594	1.0237
DTP3 (0-59 months)					
(Intercept)	4.8809	0.5682	3.9520	4.8427	6.0608
log(Dist. to urban areas)	-0.3446	0.0476	-0.4393	-0.3428	-0.2518
log(Travel time)	-0.2268	0.0621	-0.3418	-0.2288	-0.1174
log(MODIS net primary production)	0.1251	0.1662	-0.2045	0.1311	0.4322
log(Pig density)	1.0645	0.3018	0.4567	1.0792	1.6079
Spatial decay (ϕ_3)*	0.4222	0.2718	0.1690	0.2969	1.0543
Cross covariance parameters $\boldsymbol{C}_w$ for DTP1-3**					
$\boldsymbol{C}_{w(DTP1)}$	17.9196	8.0087	6.6991	18.5309	32.3521
$\boldsymbol{C}_{w(DTP1,DTP2)}$	17.7897	7.6717	7.3612	17.9730	31.4266
$\boldsymbol{C}_{w(DTP1,DTP3)}$	17.4556	7.5249	7.0439	18.4160	31.6490

$C_{w(DTP2)}$	17.9630	7.6047	7.5227	17.0508	31.9146
$C_{w(DTP2,DTP3)}$	17.5425	7.3745	7.2621	18.0415	31.5696
$C_{w(DTP3)}$	17.3890	7.4223	7.0977	17.4387	31.8864

*These correspond to effective ranges of 350 km, 1098 km, 896 km and 788 km, respectively.

**The estimated values here correspond to correlations of 0.99, 0.99 and 0.99 between DTP1 and 2, DTP1 and 3 and DTP2 and 3, respectively.

Supplementary Table 5. Estimates of the parameters of the fitted models for DRC. Reported are the posterior means, standard deviations (SD) and quantiles (2.5%, 50% and 97.5%) of the regression coefficients and the parameters of the spatial random effects.

Parameter	Mean	SD	2.5%	50%	97.5%
MCV (9-59 months)					
(Intercept)	5.3244	0.5899	4.1988	5.5107	6.1019
log(Dist. to urban areas)	-0.1725	0.0288	-0.2274	-0.1695	-0.1280
log(Cattle density)	0.3403	0.0787	0.1913	0.3381	0.4883
Temperature	-0.0124	0.0022	-0.0155	-0.0125	-0.0075
log(Pig density)	-0.0044	0.0866	-0.1929	-0.0020	0.1599
Aridity	0.1195	0.3854	-0.8509	0.2040	0.6129
Partial sill (σ^2)	2.2796	0.4218	1.6287	2.2142	3.2762
Spatial decay (ϕ)*	0.8480	0.1378	0.5531	0.8543	1.0850
DTP1 (0-59 months)					
(Intercept)	6.4043	0.5198	5.3612	6.4525	7.2008
log(Dist. to urban areas)	-0.1203	0.0255	-0.1735	-0.1186	-0.0741
Temperature	-0.0159	0.0023	-0.0190	-0.0162	-0.0108
log(Cattle density)	0.2582	0.0785	0.1077	0.2578	0.4078
Slope	-0.0415	0.0136	-0.0682	-0.0417	-0.0148
MODIS net primary production	0.1898	0.0671	0.0261	0.1917	0.3148
Spatial decay (ϕ_1)*	0.1756	0.0088	0.1663	0.1729	0.1989
DTP2 (0-59 months)					
(Intercept)	2.9825	0.7742	1.4362	3.2558	3.8154
log(Dist. to urban areas)	-0.0716	0.0203	-0.1065	-0.0736	-0.0295
Temperature	-0.0059	0.0030	-0.0095	-0.0069	0.0003
log(Cattle density)	0.1637	0.0710	0.0269	0.1636	0.3059
Slope	-0.0353	0.0125	-0.0594	-0.0356	-0.0101
MODIS net primary production	0.1900	0.0568	0.0897	0.1867	0.3152
Spatial decay (ϕ_2)*	1.0868	0.0238	1.0218	1.0939	1.1094
DTP3 (0-59 months)					

(Intercept)	2.9801	0.5840	2.0848	2.9592	4.2438
log(Dist. to urban areas)	-0.0637	0.0178	-0.0988	-0.0634	-0.0302
Temperature	-0.0103	0.0022	-0.0145	-0.0103	-0.0060
log(Cattle density)	0.2498	0.0609	0.1294	0.2499	0.3652
Slope	-0.0458	0.0125	-0.0708	-0.0454	-0.0227
MODIS net primary production	0.1471	0.0484	0.0543	0.1460	0.2444
Spatial decay (ϕ_3)*	0.2125	0.0500	0.1670	0.1957	0.3606
Cross covariance parameters $\boldsymbol{C}_w$ for DTP1-3**					
$\boldsymbol{C}_{w(DTP1)}$	12.5472	1.5773	9.3784	12.5555	15.6846
$\boldsymbol{C}_{w(DTP1,DTP2)}$	13.1156	1.4979	10.0308	13.0834	15.9053
$\boldsymbol{C}_{w(DTP1,DTP3)}$	12.1559	1.4530	9.0416	12.1270	14.8239
$\boldsymbol{C}_{w(DTP2)}$	13.9595	1.6366	10.8467	13.9638	17.1766
$\boldsymbol{C}_{w(DTP2,DTP3)}$	13.0465	1.5448	9.8739	13.0894	15.8690
$\boldsymbol{C}_{w(DTP3)}$	12.5141	1.6681	9.1015	12.5336	15.7276

*These correspond to effective ranges of 392 km, 1894 km, 306 km and 1565 km, respectively.

**The estimated values here correspond to correlations of 0.99, 0.97 and 0.99 between DTP1 and 2, DTP1 and 3 and DTP2 and 3, respectively.

Supplementary Table 6. Estimates of the parameters of the fitted models for Cambodia. Reported are the posterior means, standard deviations (SD) and quantiles (2.5%, 50% and 97.5%) of the regression coefficients and the parameters of the spatial random effects.

Parameter	Mean	SD	2.5%	50%	97.5%
MCV (9-59 months)					
(Intercept)	3.733786	0.654884	2.682752	3.684607	5.001571
log (travel time)	-0.12775	0.080679	-0.30483	-0.11762	0.006736
log (population density)	0.111343	0.046866	0.024422	0.110788	0.205731
log (distance to residential areas)	-0.0331	0.0485	-0.14257	-0.02819	0.057496
log (distance to infrastructures)	-0.17493	0.057194	-0.26908	-0.18842	-0.06486
Partial sill (σ^2)	0.432501	0.169902	0.201971	0.399369	0.81543
Spatial decay (ϕ)*	3.180514	1.294416	0.916442	3.130863	5.882994
DTP1 (0-59 months)					
(Intercept)	4.0316	0.5449	3.0542	4.0229	5.0568
log(Travel time)	-0.1108	0.0713	-0.2456	-0.1115	0.0245
MODIS net primary production	0.0000	0.0001	-0.0001	0.0000	0.0001
log(Slope)	0.4429	0.1631	0.1233	0.4432	0.7631
log(Distance to places)	-0.1841	0.0664	-0.3269	-0.1733	-0.0868

Spatial decay (ϕ_1)*	1.2160	0.3703	0.7000	1.1727	2.0645
DTP2 (0-59 months)					
(Intercept)	3.8322	0.3951	3.0654	3.7959	4.6126
log(Travel time)	-0.1331	0.0611	-0.2418	-0.1354	-0.0010
MODIS net primary production	0.0000	0.0000	-0.0001	0.0000	0.0001
log(Slope)	0.3654	0.1328	0.1148	0.3639	0.6333
log(Distance to places)	-0.2254	0.0487	-0.3206	-0.2193	-0.1410
Spatial decay (ϕ_2)*	5.4358	1.7235	0.7030	6.1478	6.5876
DTP3 (0-59 months)					
(Intercept)	3.0577	0.3157	2.5272	3.0424	3.6973
log(Travel time)	-0.0660	0.0513	-0.1702	-0.0653	0.0349
MODIS net primary production	0.0000	0.0000	-0.0001	0.0000	0.0001
log(Slope)	0.3408	0.1205	0.1116	0.3384	0.5859
log(Distance to places)	-0.1960	0.0398	-0.2868	-0.1925	-0.1214
Spatial decay (ϕ_3)*	4.8940	1.5994	0.7836	5.4269	6.5625
Cross covariance parameters $\boldsymbol{C}_w$ for DTP1-3**					
$\boldsymbol{C}_{w(DTP1)}$	1.6452	0.5849	0.8525	1.5436	2.9619
$\boldsymbol{C}_{w(DTP1,DTP2)}$	1.4912	0.4946	0.7866	1.4064	2.7079
$\boldsymbol{C}_{w(DTP1,DTP3)}$	1.3004	0.4405	0.7083	1.2268	2.2490
$\boldsymbol{C}_{w(DTP2)}$	1.5909	0.5252	0.8302	1.4983	2.8534
$\boldsymbol{C}_{w(DTP2,DTP3)}$	1.3261	0.4302	0.7042	1.2617	2.3556
$\boldsymbol{C}_{w(DTP3)}$	1.2416	0.4038	0.6484	1.1972	2.1919

*These correspond to effective ranges of 104 km, 274 km, 61 km and 68 km, respectively.
**The estimated values here correspond to correlations of 0.92, 0.91 and 0.94 between DTP1 and 2, DTP1 and 3 and DTP2 and 3, respectively.

Supplementary Table 7. Estimates of the parameters of the fitted models for Mozambique. Reported are the posterior means, standard deviations (SD) and quantiles (2.5%, 50% and 97.5%) of the regression coefficients and the parameters of the spatial random effects.

Parameter	Mean	SD	2.5%	50%	97.5%
MCV (9-59 months)					
(Intercept)	1.3506	0.9240	-0.0412	1.2366	3.0501
log (travel time)	-0.1647	0.0335	-0.2272	-0.1651	-0.0977
Precipitation	0.0080	0.0048	0.0007	0.0074	0.0181
Evapotranspiration	0.0002	0.0004	-0.0005	0.0003	0.0009
log (MODIS net primary production)	0.2934	0.2083	-0.0900	0.2887	0.7253
Partial sill (σ^2)	0.9460	0.2482	0.5531	0.9145	1.5197
Spatial decay (ϕ)*	1.4859	0.3482	0.8248	1.4746	2.1948
DTP1 (0-59 months)					

(Intercept)	5.4540	0.6630	4.2534	5.3670	6.7349
Precipitation	-0.0034	0.0043	-0.0101	-0.0041	0.0071
log(Distance to highways)	-0.2591	0.0335	-0.3215	-0.2604	-0.1923
log(Slope)	0.1299	0.0899	-0.0518	0.1315	0.3066
Evapotranspiration	-0.0003	0.0003	-0.0009	-0.0003	0.0001
Spatial decay (ϕ_1)*	0.5759	0.0832	0.4776	0.5563	0.7842
DTP2 (0-59 months)					
(Intercept)	4.8177	0.4541	3.9009	4.8152	5.6238
Precipitation	-0.0065	0.0033	-0.0123	-0.0068	0.0005
log(Distance to highways)	-0.2226	0.0320	-0.2847	-0.2231	-0.1615
log(Slope)	0.1812	0.0806	0.0268	0.1779	0.3429
Evapotranspiration	-0.0015	0.0004	-0.0023	-0.0015	-0.0009
Spatial decay (ϕ_2)*	3.0422	0.4428	1.1976	3.1678	3.2959
DTP3 (0-59 months)					
(Intercept)	4.1460	0.4909	3.1766	4.3126	4.7761
Precipitation	-0.0080	0.0037	-0.0134	-0.0087	0.0001
log(Distance to highways)	-0.1989	0.0247	-0.2493	-0.2003	-0.1509
log(Slope)	0.1900	0.0723	0.0497	0.1896	0.3439
Evapotranspiration	-0.0004	0.0002	-0.0009	-0.0004	0.0000
Spatial decay (ϕ_3)*	2.7338	0.6623	0.5949	2.9798	3.2852
Cross covariance parameters $\boldsymbol{C}_w$ for DTP1-3**					
$\boldsymbol{C}_{w(DTP1)}$	1.8554	0.4229	1.0724	1.8301	2.7564
$\boldsymbol{C}_{w(DTP1,DTP2)}$	1.6880	0.3736	1.0290	1.6484	2.5068
$\boldsymbol{C}_{w(DTP1,DTP3)}$	1.4787	0.3172	0.8994	1.4525	2.1742
$\boldsymbol{C}_{w(DTP2)}$	1.6999	0.4083	1.0379	1.6413	2.6282
$\boldsymbol{C}_{w(DTP2,DTP3)}$	1.4509	0.3329	0.8905	1.4070	2.2056
$\boldsymbol{C}_{w(DTP3)}$	1.3514	0.3138	0.8266	1.3070	2.0310

*These correspond to effective ranges of 224 km, 578 km, 109 km and 122 km, respectively.

**The estimated values here correspond to correlations of 0.95, 0.93 and 0.96 between DTP1 and 2, DTP1 and 3 and DTP2 and 3, respectively.

Supplementary Table 8: Measles Supplementary Immunization Activities (SIAs) occurring within the reference period of the DHS surveys analyzed

Country	Most recent DHS date	Number and extent of coverage of relevant measles SIAs	% reached
DRC	November 2013 - February 2014	25 (2 national and 23 subnational)	>= 93%
Ethiopia	January – June 2016	5 (2 national, 2 subnational, 1 unknown)	>=96%
Mozambique	June – November 2011	2 (national)	>=102%
Nigeria	February – June 2013	5 (2 national and 3 subnational)	26% and >=97%
Cambodia	June – December 2014	3 (national)	>=100%

[a]Source: http://www.who.int/immunization/monitoring_surveillance/data/en/

Supplementary Table 9: Regional (or administrative level 1) estimates of the coverage of DTP1-3 in children aged under five years and the dropouts between these doses in all five study countries

**Nigeria**								
Admin unit	Number of U5s	DTP1<br>% Vaccinated	DTP2<br>% Vaccinated	DTP3<br>% Vaccinated	DTP1<br>Number unvaccinated (zero-dose children)	Dropouts between DTP1 and DTP2	Dropouts between DTP2 and DTP3	Dropouts between DTP1 and DTP3
North Central	4,888,918	50.64	43.09	34.42	2,413,373	369,148	423,820	792,968
North East	4,679,566	31.57	25.41	18.75	3,202,380	288,206	311,444	599,650
North West	9,425,566	19.35	15.57	12.06	7,601,596	356,043	330,736	686,779
South East	2,613,667	82.53	76.96	68.38	456,605	145,562	224,161	369,723
South South	3,410,288	76.18	69.82	59.52	812,173	217,165	351,228	568,393
South West	4,798,175	71.37	61.07	50.23	1,373,647	494,306	520,247	1,014,554
**Cambodia**								

Admin unit	Number of U5s	DTP1 % Vaccinated	DTP2 % Vaccinated	DTP3 % Vaccinated	DTP1 Number unvaccinated (zero-dose children)	Dropouts between DTP1 and DTP2	Dropouts between DTP2 and DTP3	Dropouts between DTP1 and DTP3
Bântéay Méanchey	89,608	93.24	86.94	83.32	6,058	5,649	3,244	8,893
Batdâmbâng	135,666	91.50	84.42	80.60	11,528	9,602	5,192	14,794
Kâmpôt	76,202	85.76	74.55	72.03	10,849	8,543	1,923	10,466
Kâmpóng Cham	120,966	90.56	85.14	81.90	11,419	6,561	3,910	10,471
Kâmpóng Chhnang	61,553	90.28	81.97	78.33	5,980	5,116	2,240	7,356
Kâmpóng Spœ	92,090	88.92	80.80	77.32	10,201	7,477	3,210	10,687
Kâmpóng Thum	82,531	91.85	85.02	81.98	6,729	5,636	2,504	8,140
Kândal	166,062	92.05	86.98	83.56	13,198	8,421	5,685	14,106
Kaôh Kong	18,617	89.53	80.71	78.67	1,949	1,642	379	2,021
Kep	4,666	83.26	70.60	67.35	781	591	151	742
Krâchéh	41,927	84.20	73.73	71.24	6,626	4,388	1,046	5,434
Krong Pailin	9,395	94.31	88.65	85.71	535	531	277	808

Krong Preah Sihanouk	25,783	90.39	82.56	79.70	2,477	2,019	736	2,756
Môndól Kiri	7,962	75.63	63.08	62.28	1,940	999	63	1,063
Otdar Mean Chey	22,693	89.86	82.04	79.18	2,301	1,774	648	2,423
Phnom Penh	180,972	95.43	92.59	88.52	8,277	5,138	7,359	12,497
Pouthisat	52,048	92.81	85.86	82.90	3,744	3,618	1,539	5,157
Preah Vihéar	22,496	85.78	75.52	73.13	3,199	2,308	537	2,845
Prey Vêng	124,085	90.68	84.64	81.61	11,566	7,488	3,760	11,248
Rôtânôkiri	19,823	75.13	60.90	60.04	4,931	2,821	170	2,991
Siemréab	120,560	91.00	83.94	80.00	10,846	8,522	4,748	13,270
Stœng Trêng	14,623	86.40	74.98	72.39	1,988	1,670	379	2,049
Svay Rieng	63,360	90.07	84.30	81.68	6,292	3,656	1,660	5,316
Takêv	110,867	90.18	82.20	79.32	10,888	8,844	3,191	12,035
Tbong Khmum	99,130	86.33	77.59	74.60	13,555	8,656	2,969	11,625
**Mozambique**								
Admin unit	Number of U5s	DTP1 % Vaccinated	DTP2 % Vaccinated	DTP3 % Vaccinated	DTP1 Number unvaccinated	Dropouts between DTP1 and DTP2	Dropouts between DTP2 and DTP3	Dropouts between DTP1 and DTP3

					(zero-dose children)			
Cabo Delgado	335,332	85.72	77.72	67.09	47,891	26,829	35,631	62,460
Gaza	207,288	92.00	87.00	79.09	16,582	10,360	16,405	26,766
Inhambane	268,323	91.46	86.01	76.91	22,920	14,608	24,423	39,031
Manica	325,458	87.59	80.72	71.68	40,374	22,366	29,445	51,811
Maputo City	451,606	93.10	89.09	83.89	31,175	18,090	23,482	41,572
Maputo	108,880	95.04	91.95	86.91	5,405	3,357	5,488	8,845
Nampula	860,995	82.75	74.01	63.14	148,494	75,263	93,573	168,836
Niassa	276,241	84.70	77.96	68.06	42,271	18,624	27,329	45,953
Sofala	356,528	91.24	85.03	76.08	31,236	22,141	31,905	54,046
Tete	418,915	81.45	74.31	64.98	77,692	29,915	39,111	69,026
Zambezia	835,385	73.38	63.49	52.08	222,415	82,546	95,358	177,904
**DRC**								
Admin unit	Number of U5s	DTP1 % Vaccinated	DTP2 % Vaccinated	DTP3 % Vaccinated	DTP1 Number unvaccinated (zero-dose children)	Dropouts between DTP1 and DTP2	Dropouts between DTP2 and DTP3	Dropouts between DTP1 and DTP3

Équateur	1,479,489	57.85	45.93	31.34	623,600	176,395	215,754	392,149
Bandundu	1,527,121	75.57	67.54	51.76	373,026	122,655	241,008	363,663
Bas-Congo	554,838	86.21	82.28	72.95	76,520	21,784	51,793	73,577
Kasaï-Occidental	1,200,500	75.21	64.44	45.83	297,591	129,251	223,467	352,717
Kasaï-Oriental	1,446,431	57.66	46.12	31.11	612,376	166,972	217,104	384,076
Katanga	1,848,649	54.98	47.50	36.07	832,242	138,290	211,327	349,617
Kinshasa City	991,289	80.57	74.91	63.80	192,635	56,031	110,189	166,220
Kivu	2,111,920	71.89	64.55	50.19	593,576	155,066	303,375	458,441
Orientale	2,562,693	63.19	52.18	36.24	943,350	282,201	408,504	690,705
**Ethiopia**								
Admin unit	Number of U5s	DTP1 % Vaccinated	DTP2 % Vaccinated	DTP3 % Vaccinated	DTP1 Number unvaccinated (zero-dose children)	Dropouts between DTP1 and DTP2	Dropouts between DTP2 and DTP3	Dropouts between DTP1 and DTP3

Addis Ababa	596,231	90.16	85.24	78.63	58,641	29,380	39,368	68,748
Afar	293,550	29.70	19.65	14.38	206,352	29,508	15,471	44,979
Amhara	3,912,029	69.73	58.29	46.27	1,184,157	447,446	470,410	917,856
Benshangul-Gumaz	158,668	72.54	65.10	54.74	43,576	11,802	16,430	28,232
Dire Dawa	80,912	76.41	64.99	53.89	19,088	9,239	8,977	18,217
Gambela Peoples	67,853	53.23	41.99	29.67	31,735	7,624	8,362	15,986
Harari People	35,912	60.30	46.02	35.59	14,255	5,130	3,745	8,875
Oromia	5,364,868	57.73	45.67	35.44	2,267,627	646,912	549,158	1,196,071
Somali	823,850	24.75	14.43	9.73	619,924	85,050	38,698	123,748
Southern Nations, Nationalities and Peoples	2,941,194	66.19	55.18	43.14	994,376	323,886	354,101	677,987
Tigray	902,113	80.27	74.23	66.69	177,992	54,470	68,008	122,479

Supplementary Table 10: Percentages of surveyed children aged ≤ 1 month and ≤ 3 months in the study countries

Country	Percentage of under 5s aged ≤ 1 month	Percentage of under 5s aged ≤ 3 months
Ethiopia	4.9	10.9
Mozambique	3.1	6.7
Cambodia	2.9	6.4
Nigeria	6.4	10.2
DRC	3.0	7.2

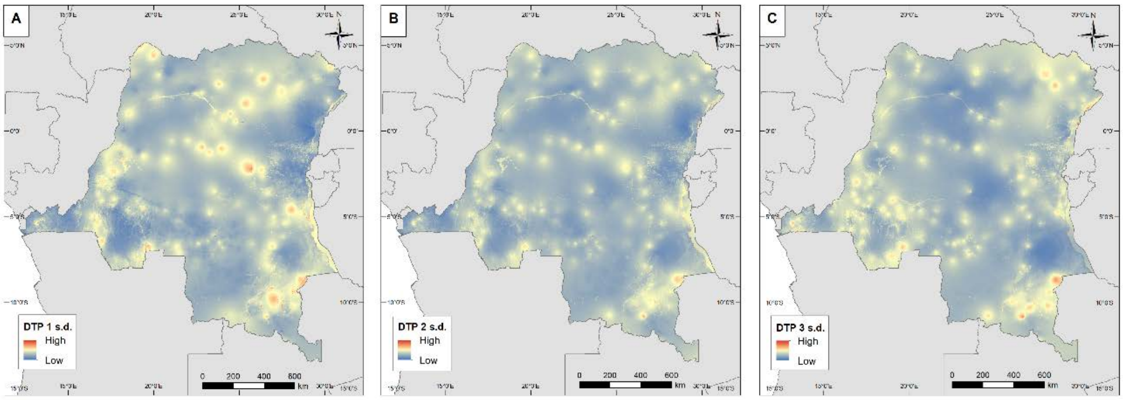

Supplementary Figure 1: (A-C) Uncertainty (standard deviation) maps for DTP doses 1, 2 and 3 vaccination coverage in children under 5 in DRC.

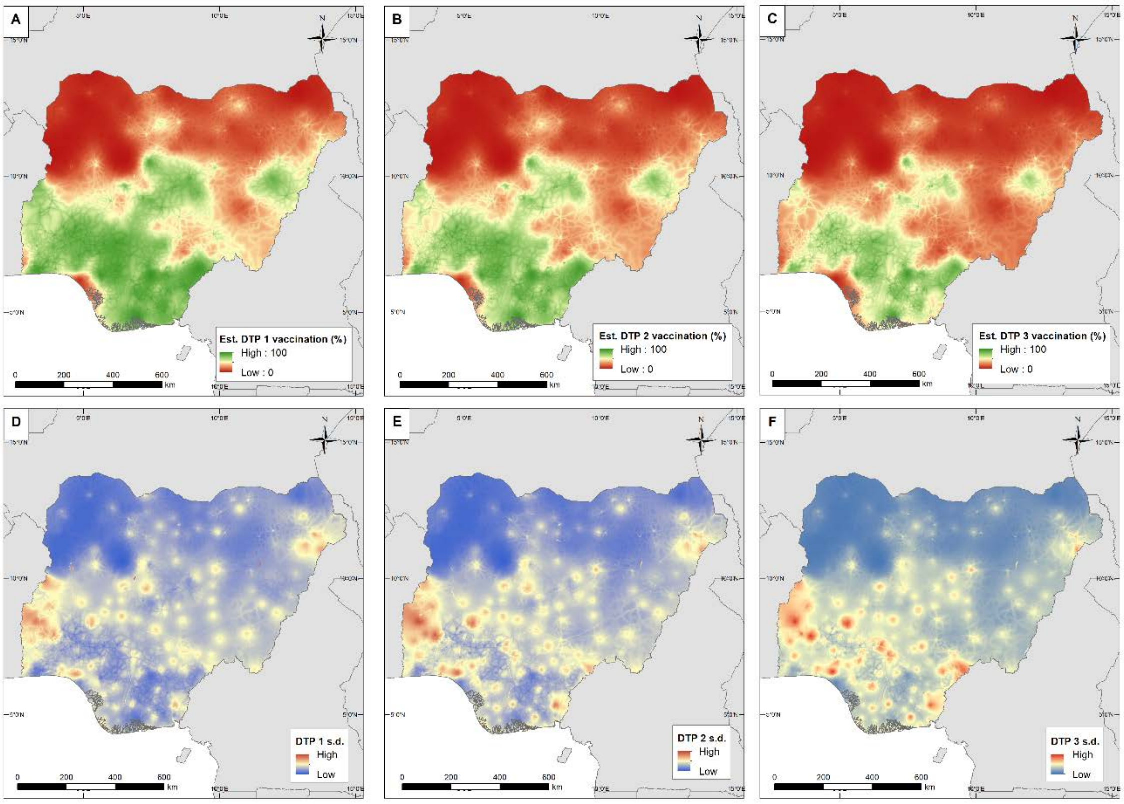

Supplementary Figure 2: (A - C) Estimated DTP doses 1, 2 and 3 vaccination coverage in children under 5 years old at 1x1 km resolution for Nigeria in 2013. (D - F) Associated uncertainty maps measured as standard deviations.

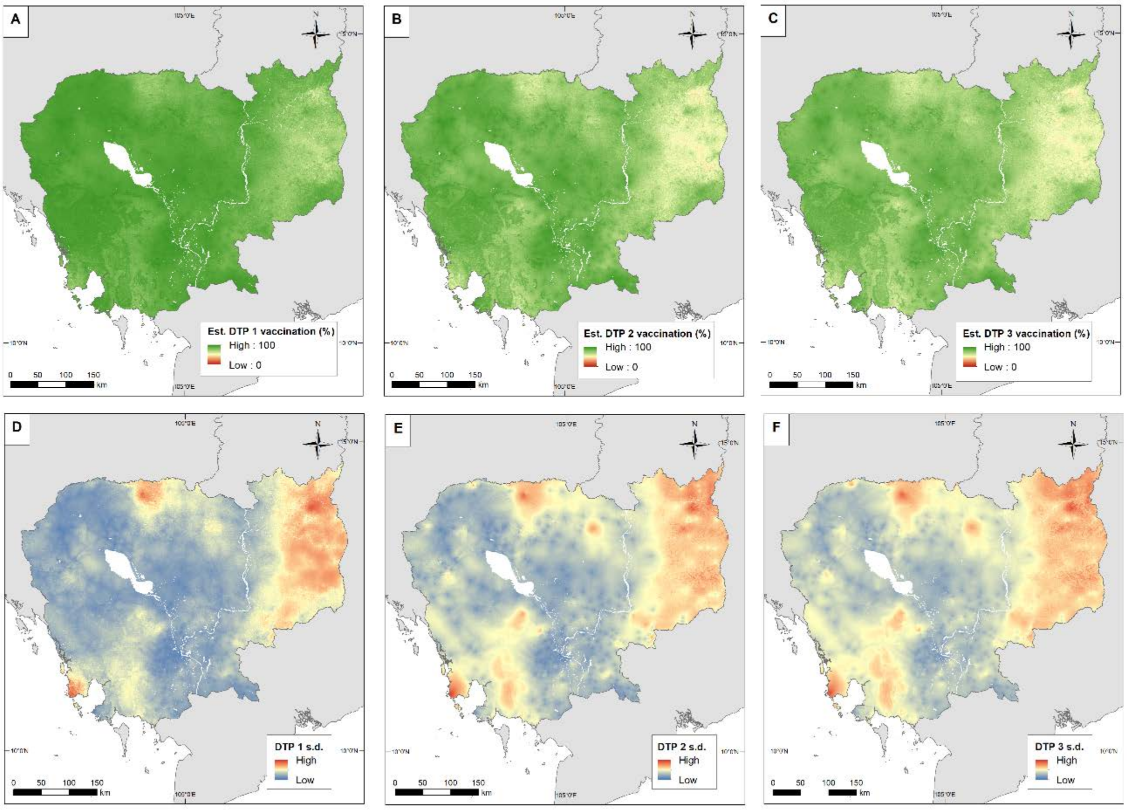

Supplementary Figure 3: (A - C) Estimated DTP doses 1, 2 and 3 vaccination coverage in children under 5 years old at 1x1 km resolution for Cambodia in 2014. (D - F) Associated uncertainty maps measured as standard deviations.

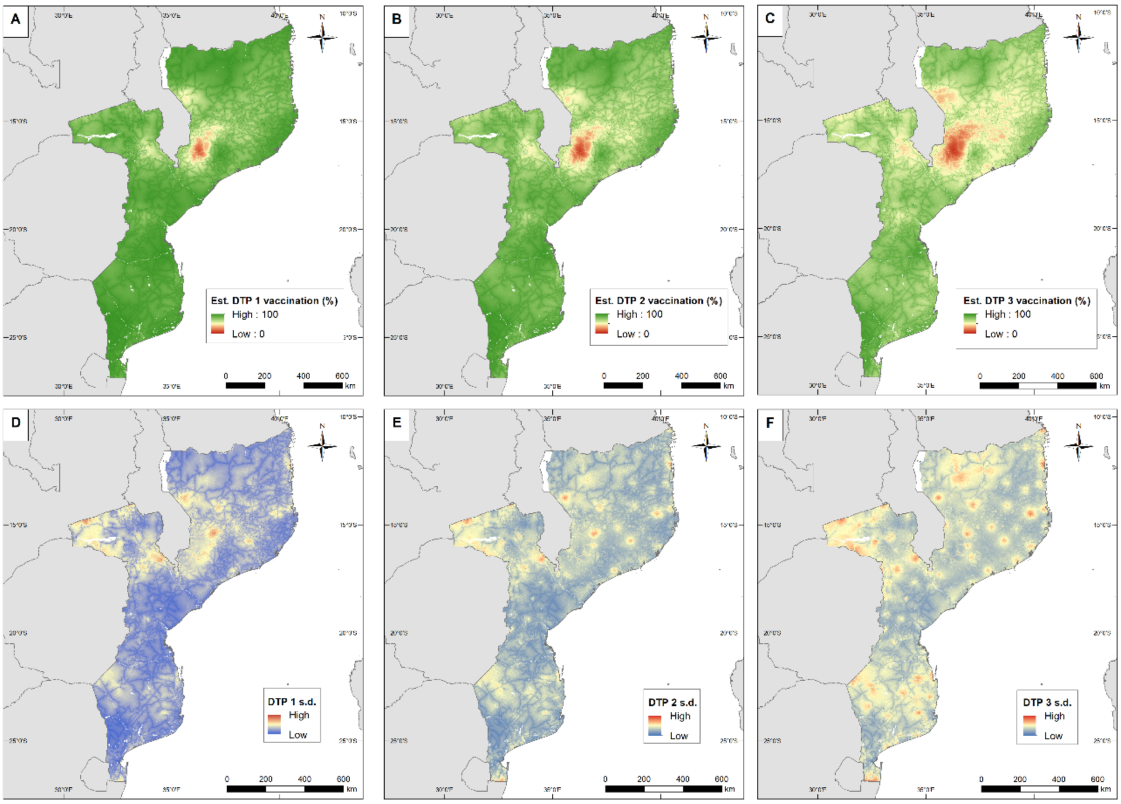

Supplementary Figure 4: (A - C) Estimated DTP doses 1, 2 and 3 vaccination coverage in children under 5 years old at 1x1 km resolution for Mozambique in 2011. (D - F) Associated uncertainty maps measured as standard deviations.

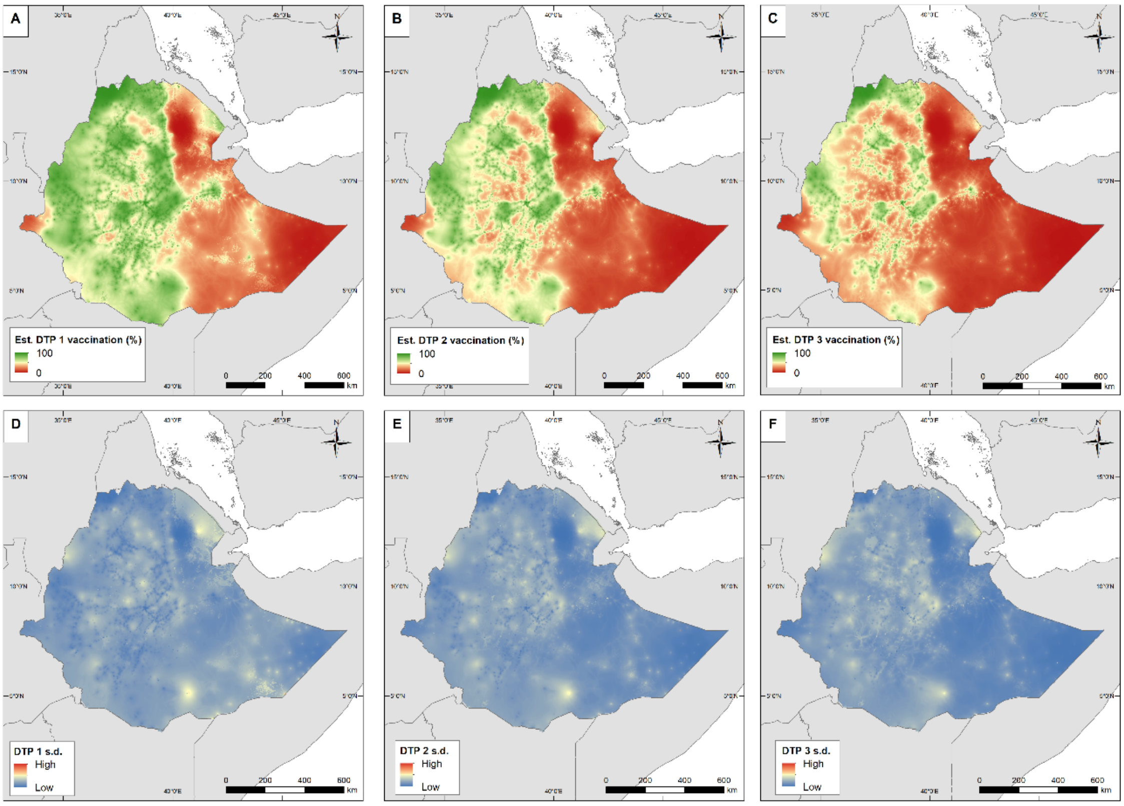

Supplementary Figure 5: (A - C) Estimated DTP doses 1, 2 and 3 vaccination coverage in children under 5 years old at 1x1 km resolution for Ethiopia in 2016. (D - F) Associated uncertainty maps measured as standard deviations.

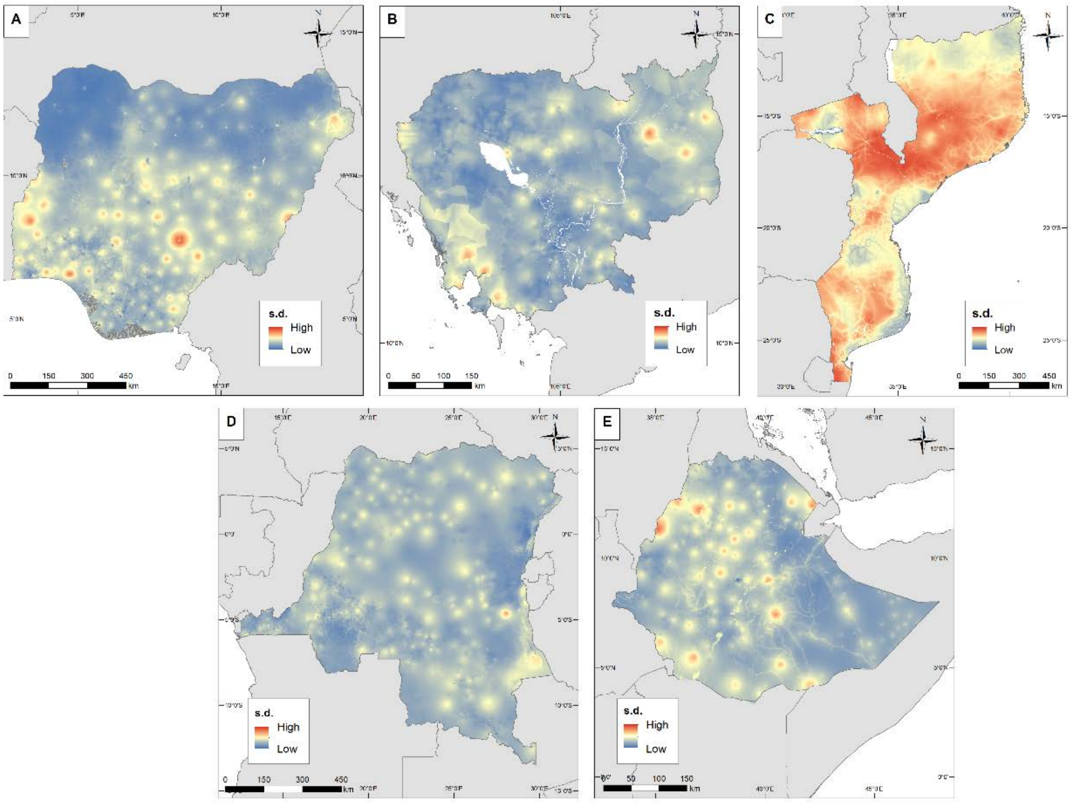

Supplementary Figure 6: Uncertainty (standard deviation) maps for measles vaccination coverage in children aged 9-59 months in (A) Nigeria, (B) Cambodia, (C) Mozambique, (D) DRC and (E) Ethiopia.

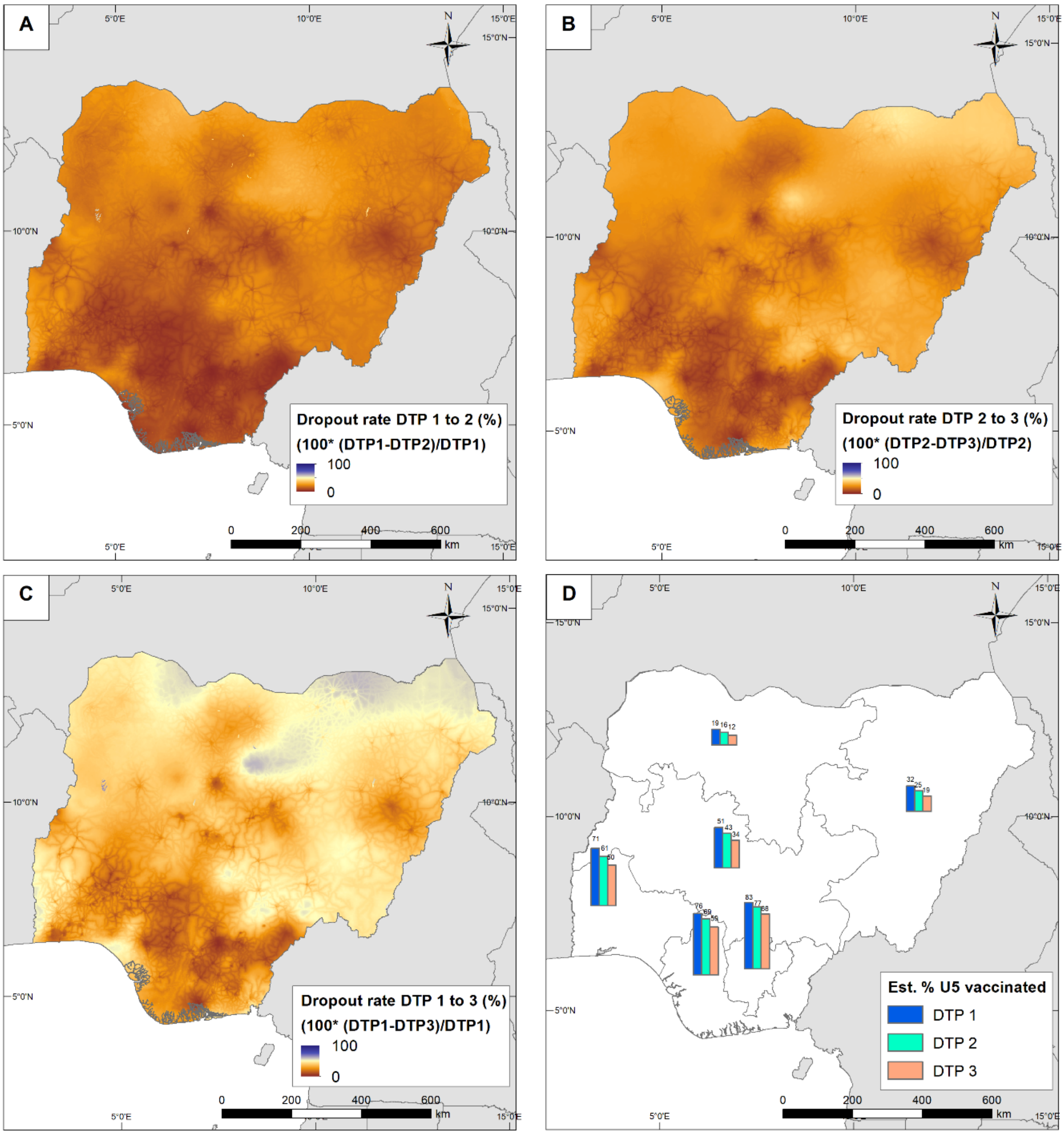

Supplementary Figure 7: (A – C) Estimated dropout rates between DTP vaccination doses 1 to 3 in children under 5 years old at 1x1 km resolution for Nigeria in 2013. The estimated percentage of children receiving each dose in each administrative unit is shown in panel (D).

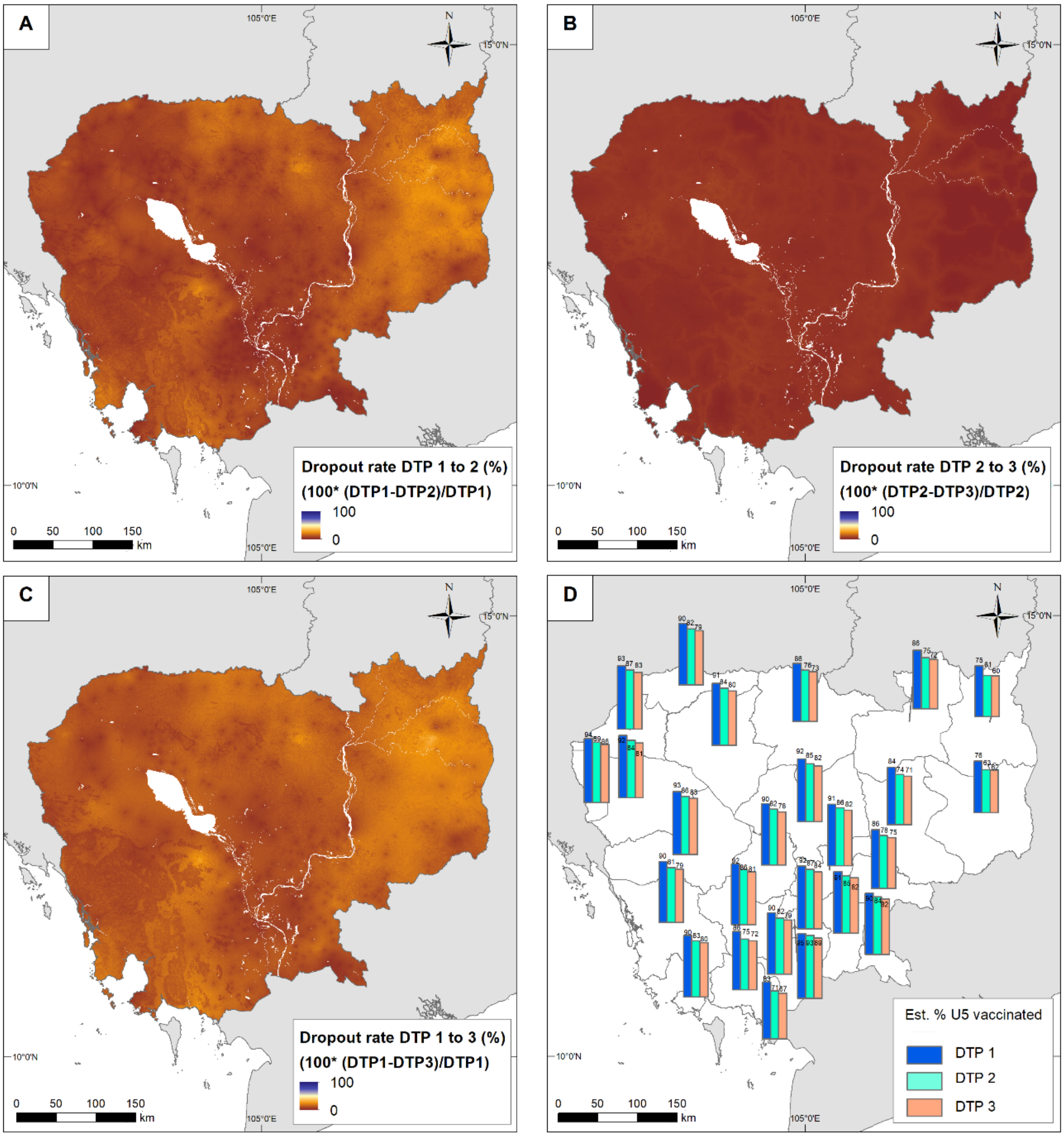

Supplementary Figure 8: (A - C) Estimated dropout rates between DTP vaccination doses 1 to 3 in children under 5 years old at 1x1 km resolution for Cambodia in 2014. The estimated percentage of children receiving each dose in each administrative unit is shown in panel (D).

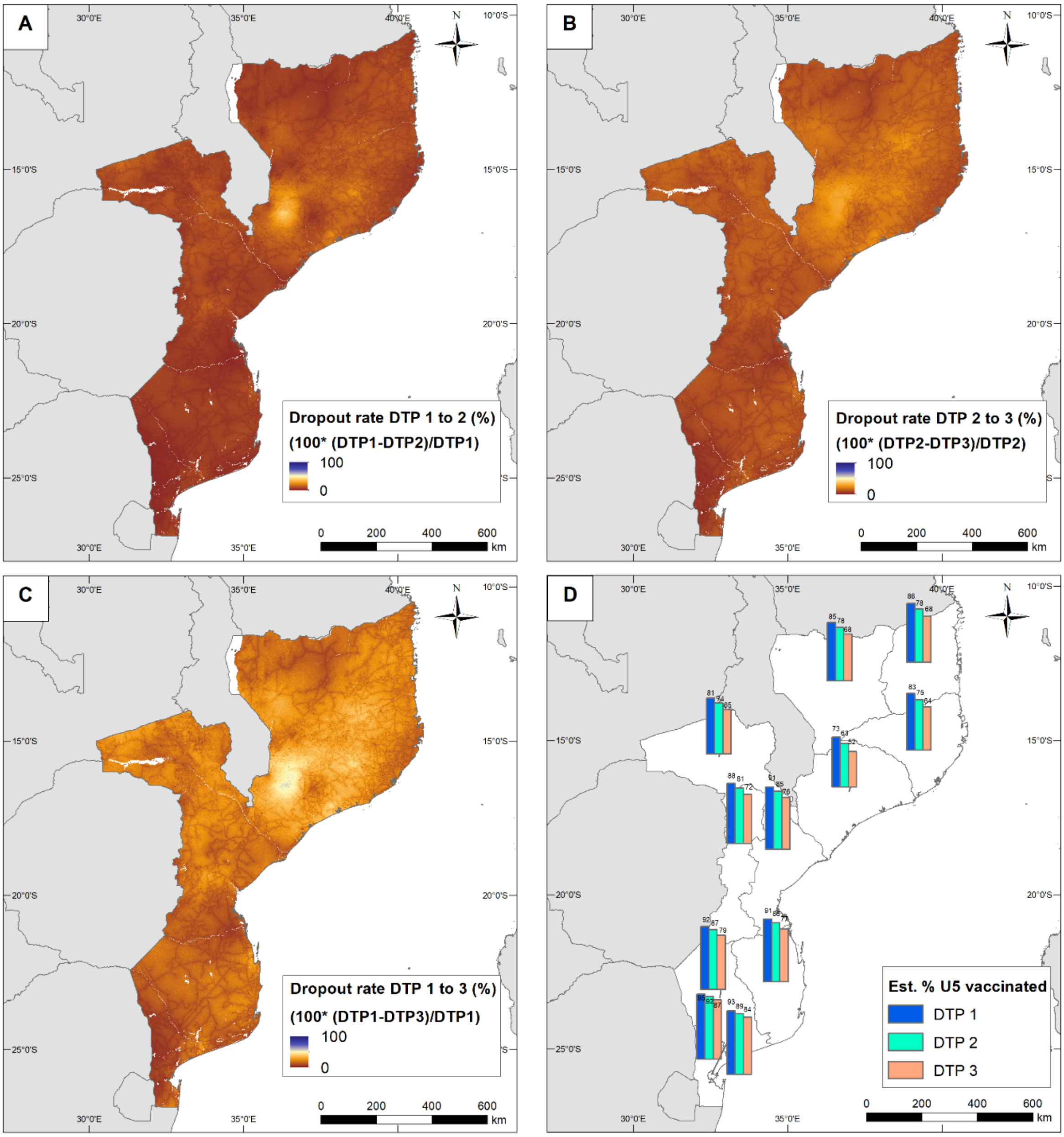

Supplementary Figure 9: (A - C) Estimated dropout rates between DTP vaccination doses 1 to 3 in children under 5 years old at 1x1 km resolution for Mozambique in 2011. The estimated percentage of children receiving each dose in each administrative unit is shown in panel (D).

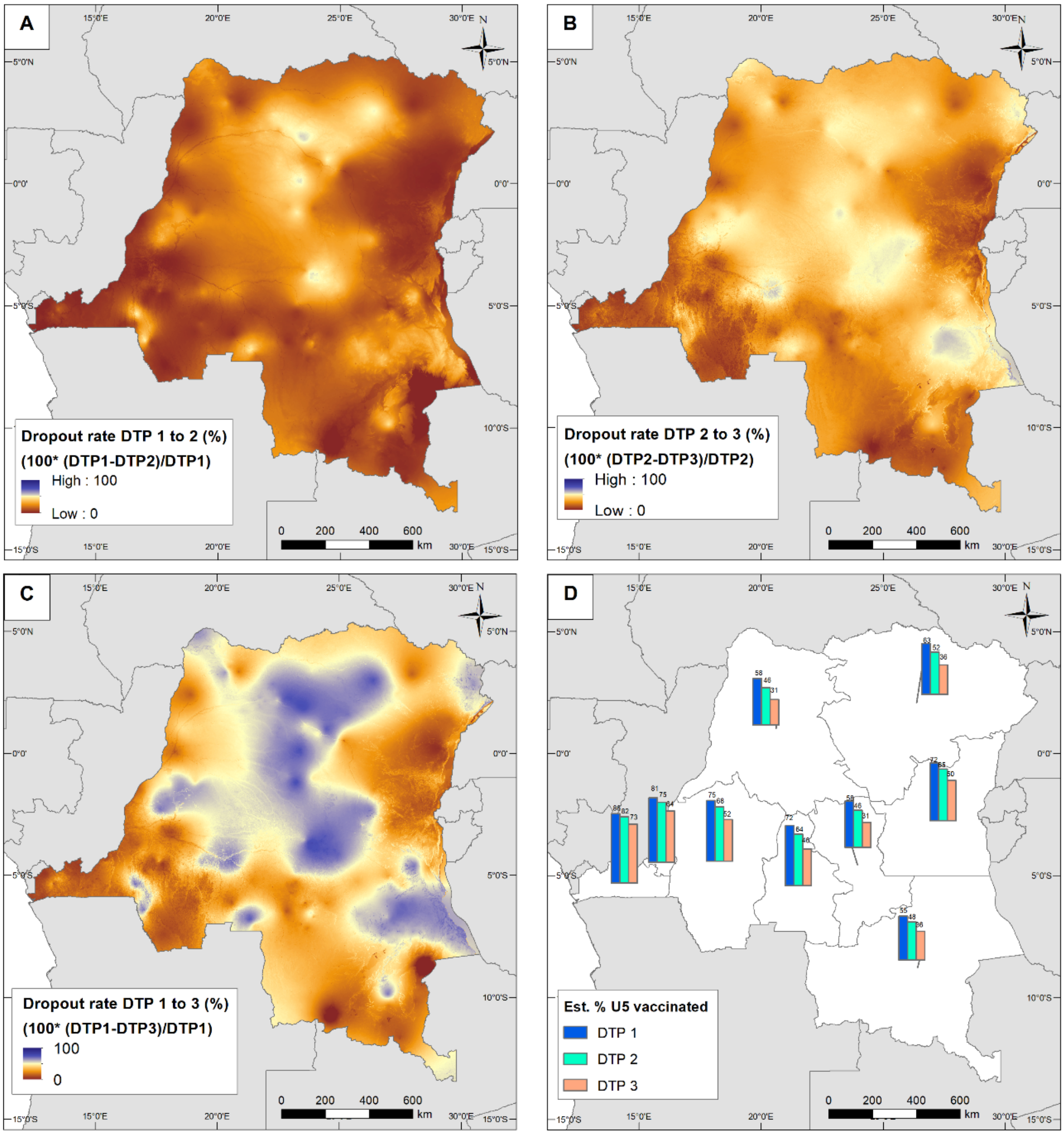

Supplementary Figure 10: (A - C) Estimated dropout rates between DTP vaccination doses 1 to 3 in children under 5 years old at 1x1 km resolution for DRC in 2013-14. The estimated percentage of children receiving each dose in each administrative unit is shown in panel (D).

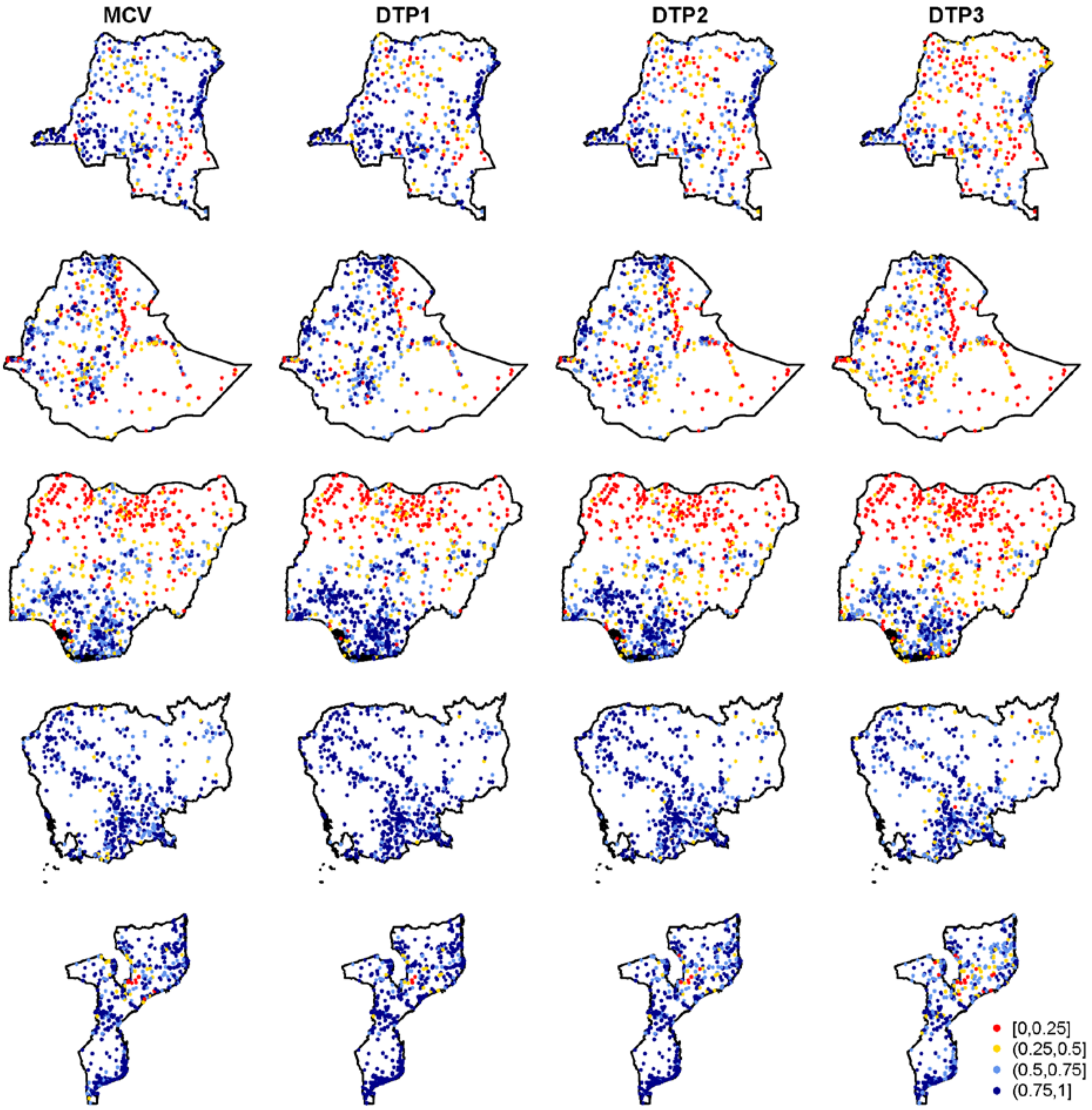

Supplementary Figure 11: DHS survey cluster locations and observed vaccination coverage for MCV (9-59 months) and DTP1-3 (0-59 months) for (top-bottom) DRC 2014, Ethiopia 2016, Nigeria 2013, Cambodia 2014, and Mozambique 2011.

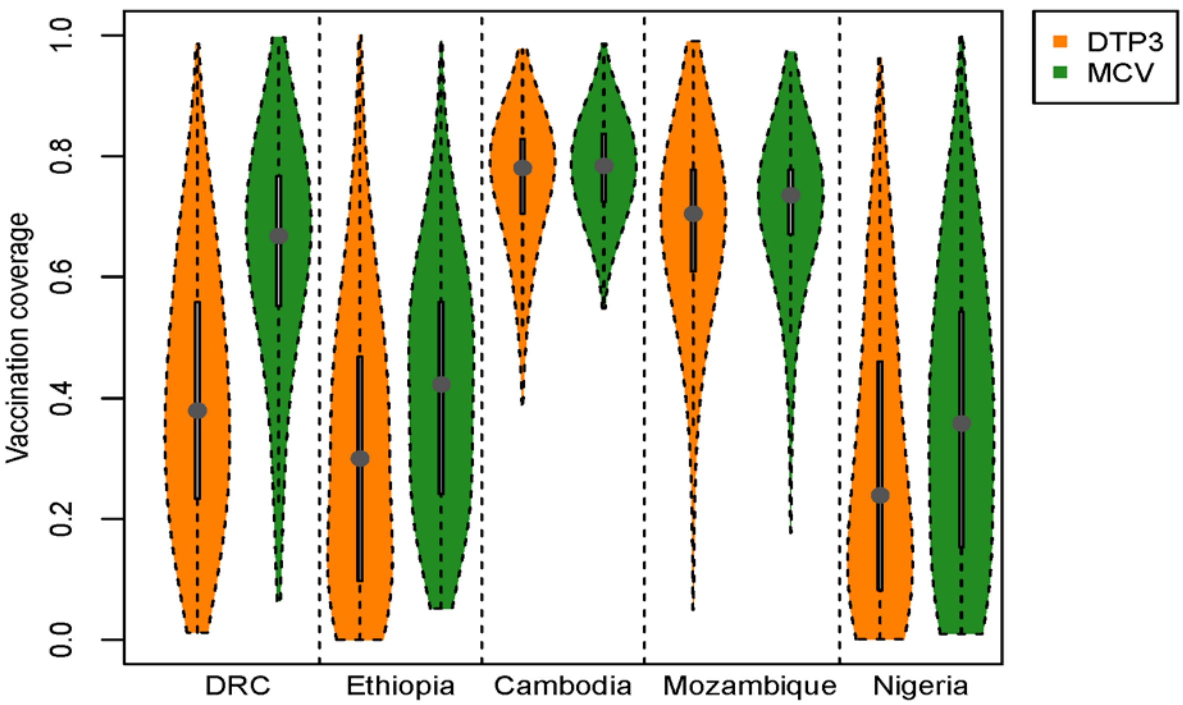

Supplementary Figure 12: Plots of the distributions of DTP3 and MCV coverage in all five study countries. The grey dots in the violin plots represent the medians of the distributions while the thick grey bars show their interquartile range.

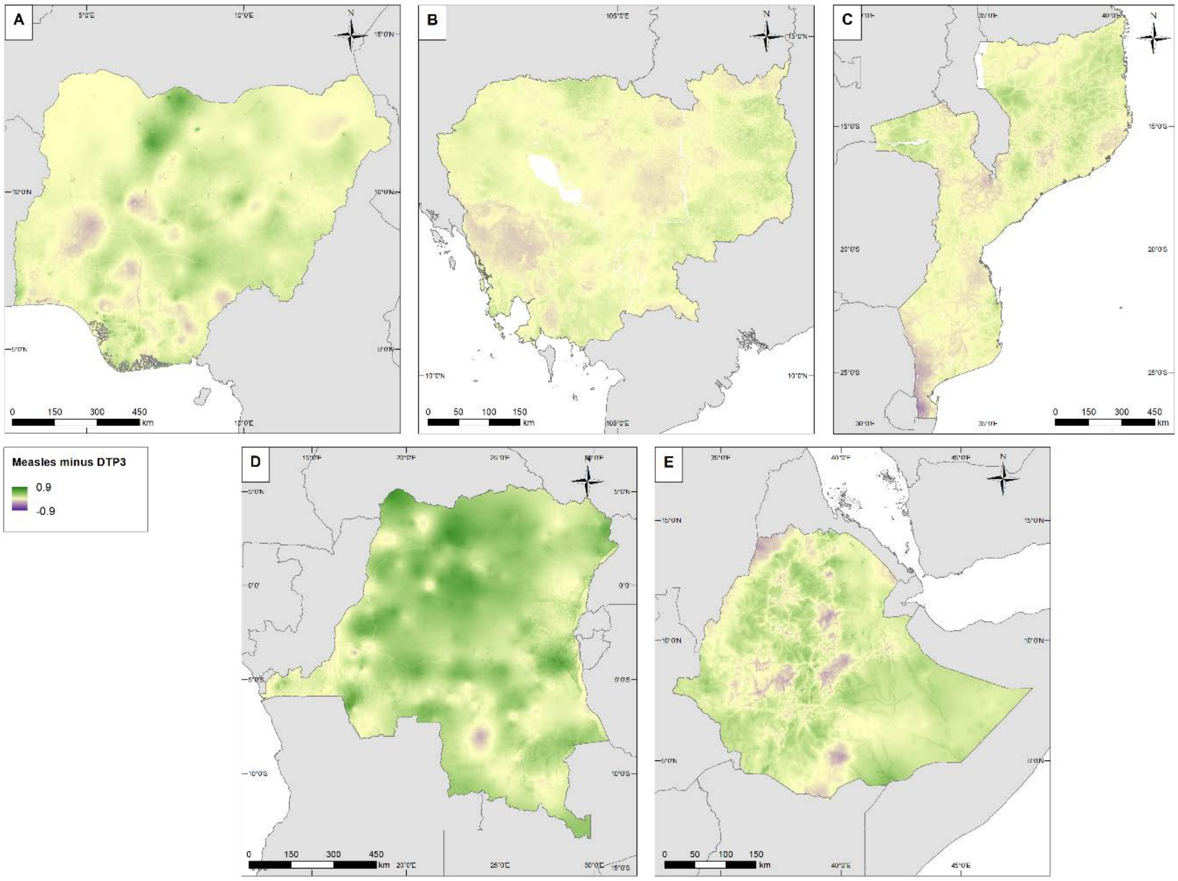

Supplementary Figure 13: Differences between predicted measles and DTP3 coverage at 1x1 km resolution for (A - E) Nigeria, Cambodia, Mozambique, DRC and Ethiopia.

Supplementary References

1. Finley AO, Banerjee S, Carlin BP. spBayes: An R Package for Univariate and Multivariate Hierarchical Point-referenced Spatial Models. *Journal of statistical software* **19**, 1-24 (2007).

2. Finley AO, Banerjee S, Ek AR, McRoberts RE. Bayesian multivariate process modeling for prediction of forest attributes. *Journal of Agricultural, Biological, and Environmental Statistics* **13**, 60 (2008).

3. Royle JA, Nychka D. An algorithm for the construction of spatial coverage designs with implementation in SPLUS. *Computers & Geosciences* **24**, 479-488 (1998).

4. Utazi CE*, et al.* High resolution age-structured mapping of childhood vaccination coverage in low and middle income countries. *Vaccine* **36**, 1583-1591 (2018).

5. Geweke J. Evaluating the accuracy of sampling-based approaches to calculating posterior moments. In: *Bayesian Statistics 4* (eds. Bernado JM, Berger JO, Dawid AP, Smith AFM). Clarendon Press (1992).
