# Supplementary material for: Mapping vaccination coverage to explore the effects of delivery mechanisms and inform vaccination strategies
Source: Nat Commun. 2019 Apr 9;10:1633. doi: 10.1038/s41467-019-09611-1 (PMC6456602; doi:10.1038/s41467-019-09611-1)
Supplement: Supplementary file 3 — Reporting Summary [file 41467_2019_9611_MOESM3_ESM.pdf]

nature research

Corresponding author(s): Dr Chigozie Utazi (c.e.utazi@soton.ac.uk)
University of Southampton

Last updated by author(s): March 10, 2019

Reporting Summary

Nature Research wishes to improve the reproducibility of the work that we publish. This form provides structure for consistency and transparency in reporting. For further information on Nature Research policies, see Authors & Referees and the Editorial Policy Checklist.

Statistics

For all statistical analyses, confirm that the following items are present in the figure legend, table legend, main text, or Methods section.

n/a	Confirmed	
☒	☐	The exact sample size (*n*) for each experimental group/condition, given as a discrete number and unit of measurement
☒	☐	A statement on whether measurements were taken from distinct samples or whether the same sample was measured repeatedly
☒	☐	The statistical test(s) used AND whether they are one- or two-sided<br>*Only common tests should be described solely by name; describe more complex techniques in the Methods section.*
☐	☒	A description of all covariates tested
☒	☐	A description of any assumptions or corrections, such as tests of normality and adjustment for multiple comparisons
☐	☒	A full description of the statistical parameters including central tendency (e.g. means) or other basic estimates (e.g. regression coefficient) AND variation (e.g. standard deviation) or associated estimates of uncertainty (e.g. confidence intervals)
☒	☐	For null hypothesis testing, the test statistic (e.g. *F*, *t*, *r*) with confidence intervals, effect sizes, degrees of freedom and *P* value noted<br>*Give P values as exact values whenever suitable.*
☐	☒	For Bayesian analysis, information on the choice of priors and Markov chain Monte Carlo settings
☒	☐	For hierarchical and complex designs, identification of the appropriate level for tests and full reporting of outcomes
☒	☐	Estimates of effect sizes (e.g. Cohen's *d*, Pearson's *r*), indicating how they were calculated

Our web collection on statistics for biologists contains articles on many of the points above.

Software and code

Policy information about availability of computer code

Data collection	No software was used for data collection. The data used were downloaded from the repositories of the Demographic and Health Surveys (DHS) program as stated in the manuscript.
Data analysis	Both the univariate and multivariate analyses conducted in the study were implemented using spBayes package in R as referenced in the paper.

For manuscripts utilizing custom algorithms or software that are central to the research but not yet described in published literature, software must be made available to editors/reviewers. We strongly encourage code deposition in a community repository (e.g. GitHub). See the Nature Research guidelines for submitting code & software for further information.

Data

Policy information about availability of data

All manuscripts must include a data availability statement. This statement should provide the following information, where applicable:

- Accession codes, unique identifiers, or web links for publicly available datasets
- A list of figures that have associated raw data
- A description of any restrictions on data availability

All data used in this work is publicly available via the sources referenced in the Methods section. These can be obtained from the authors upon request.

Field-specific reporting

Please select the one below that is the best fit for your research. If you are not sure, read the appropriate sections before making your selection.

☒ Life sciences ☐ Behavioural & social sciences ☐ Ecological, evolutionary & environmental sciences

Life sciences study design

All studies must disclose on these points even when the disclosure is negative.

Sample size	Demographic and Health Surveys (DHS) are designed to be nationally representative. All information regarding sample size determination is provided by the DHS program in the reports produced following each survey.
Data exclusions	Only survey clusters where no child was sampled were excluded from the analyses.
Replication	All analyses can be replicated using the information provided in the Methods section.
Randomization	The DHS program uses a multi-stage cluster sampling design in selecting the primary sampling units (PSUs) included in the survey.
Blinding	DHS surveys involve the administration of standard or uniform questionnaires to all participants. So blinding does not apply in this case.

Reporting for specific materials, systems and methods

We require information from authors about some types of materials, experimental systems and methods used in many studies. Here, indicate whether each material, system or method listed is relevant to your study. If you are not sure if a list item applies to your research, read the appropriate section before selecting a response.

Materials & experimental systems

n/a	Involved in the study	
☒	☐	Antibodies
☒	☐	Eukaryotic cell lines
☒	☐	Palaeontology
☒	☐	Animals and other organisms
☐	☒	Human research participants
☒	☐	Clinical data

Methods

n/a	Involved in the study	
☒	☐	ChIP-seq
☒	☐	Flow cytometry
☒	☐	MRI-based neuroimaging

Human research participants

Policy information about studies involving human research participants

Population characteristics	All relevant population characteristics were determined by the Demographic and Health Surveys (DHS) program who collected the data used in the work.
Recruitment	The study used secondary data obtained by the Demographic and Health Surveys program.
Ethics oversight	The Demographic and Health Surveys (DHS) program

Note that full information on the approval of the study protocol must also be provided in the manuscript.
